# Mapping the current status and outlook of research on noonan syndrome over the last 26 years: a bibliometric and visual analysis

**DOI:** 10.3389/fgene.2024.1488425

**Published:** 2024-12-12

**Authors:** Zhengjiu Cui, Yuanyuan Wang, Fei Luo, Juanjuan Diao, Bin Yuan

**Affiliations:** ^1^ Department of Pediatrics, Affiliated Hospital of Nanjing University of Chinese Medicine, Nanjing, China; ^2^ Department of Pediatrics, Suqian Affiliated Hospital of Nanjing University of Chinese Medicine, Suqian, China; ^3^ Department of Pediatrics, Affiliated Hospital of Shandong University of Traditional Chinese Medicine, Jinan, China

**Keywords:** noonan syndrome, bibliometric analysis, genetic mutation, trends, cooperation, web of science

## Abstract

**Background:**

Noonan syndrome (NS) is a rare group of autosomal genetic disorders. In recent years, with the exploration and development of molecular diagnostic techniques, more and more researchers have begun to pay attention to NS. However, there is still a lack of reports on the bibliometric analysis of NS worldwide. This study aims to assess the current research status and development trend of NS, to explore the research hotspots and emerging topics, and to point out the direction for future scientific research.

**Methods:**

Web of Science Core Collection was selected as the search database for bibliometric analysis of NS-related publications from 1998 to 2023. Statistical and visual analysis of the number of publications, countries, institutions, authors, journals, keywords, and references were analyzed using Citespace, VOSviewer, Scimago Graphica, and BibliometrixR.

**Results:**

A total of 2041 articles were included in this study. The United States had the highest number of publications, and Istituto Superiore di Sanità, Italy, was the institution with the highest number of publications. TARTAGLIA M was the scientist with the highest number of publications and citations. Among the journals, AMERICAN JOURNAL OF MEDICAL GENETICS PART A has the highest output, and Nature Genetics is the most frequently cited. The reference with the highest outburst intensity is Roberts AE, LANCET, 2013. the cluster diagram divides all the keywords into seven categories, with the most vigorous outburst being “of function mutations.”

**Conclusion:**

Research hotspots in the field of NS focus on the correspondence between NS genotype and phenotype and the precise diagnosis of NS. Future research efforts will explore more deeply from the perspective of long-term intervention strategies for NS. There is an urgent need to rely on significant research countries, institutions, journals, and authors to lead the construction of a more robust global collaborative network that will enhance research efficacy.

## 1 Introduction

Noonan syndrome (NS, OMIM 163950) is a rare autosomal genetic disease, most of which are dominantly inherited. However, a few recessive inheritances can also be seen, and the prevalence of NS in live births is about 0.04%–0.1% (Romano et al., 2010). NS can involve different organs in several systems of the body, and the clinical manifestations are diverse. Children with NS are mainly characterized by special facial features, short stature, thoracic deformities, and heart disease (Cirstea et al., 2010). In infants and early to middle childhood, special facial features are more pronounced, including inverted triangular faces, wide eye spacing, drooping eyes, and low ear positions, among others (Baldo et al., 2022). However, these facial features will gradually fade with age. Cardiac lesions are present in more than 80% of NS patients during the disease, with pulmonary valve stenosis being the most common (Prendiville et al., 2014). The disease can also be associated with cryptorchidism, renal malformations, psychomotor retardation, tumors, and hematologic disorders (Tartaglia et al., 2011). The pathogenesis of NS is poorly understood, and previous studies have found that approximately 80% of the onset of the disease is associated with mutations in the genes coding for the components or the regulators of the mitogen-activated protein kinase (RAS-MAPK) signaling pathway (Tartaglia and Gelb, 2005). Fifty percent of NS patients have missense mutations in PTPN11, the most common known causative gene for NS (Aria and Cuccurullo, 2017). There is no specific treatment for NS, and symptomatic treatment is still the mainstay. Although the incidence of this disease is low, based on the calculation of the vast global population, it is still a group whose number should not be underestimated. The complex symptoms and complications brought by NS cannot be cured entirely. In the long term, it seriously affects the physical and mental health of the affected children. It even shortens their life span, which bothers countless clinicians and the families of the affected children. With the advancement of science and technology and the continuous development of medicine, researchers have accumulated a certain amount of knowledge about NS and have conducted comprehensive and in-depth studies on various aspects, such as pathogenesis, diagnosis, and treatment, and have made many breakthroughs at various stages.

Bibliometrics can be traced back to the beginning of the 20th century when only mathematical methods were introduced into the literature statistics, called “Literature statistics.” In the mid-21st century, the integration of computer technology has made the theory and application of the method extensively developed. Bibliographer A. Pritchard formally put forward “bibliometrics” in 1969, marking the establishment of an independent discipline, since then it has been widely used in various fields of research (Ninkov et al., 2022). It is a product of the comprehensive intersection of mathematics, statistics, and bibliography, which is able to quantitatively analyze all knowledge carriers and visually present detailed information such as countries, institutions, journals, authors, keywords, and references through visualization methods and finds research hotspots and cutting-edge directions in the field, which is conducive to the search for the theme for the next step of the research. Currently, bibliometric research is viral and significant in the medical field (Kokol et al., 2021).

To the best of our knowledge, bibliometric analysis of NS has yet to be published in major global databases as of now. In this study, we mainly used Citespace, VOSviewer, Scimago Graphica, and Bibliometrics program packages to conduct a comprehensive analysis of published high-quality papers on NS between 1998 and 2023 to explore the current status and hotspots of global research in the field, to explore the future research trends, and to provide an all-rounded for the continuation of the research on NS.

## 2 Materials and methods

### 2.1 Data sources and search strategies

**FIGURE 1:**
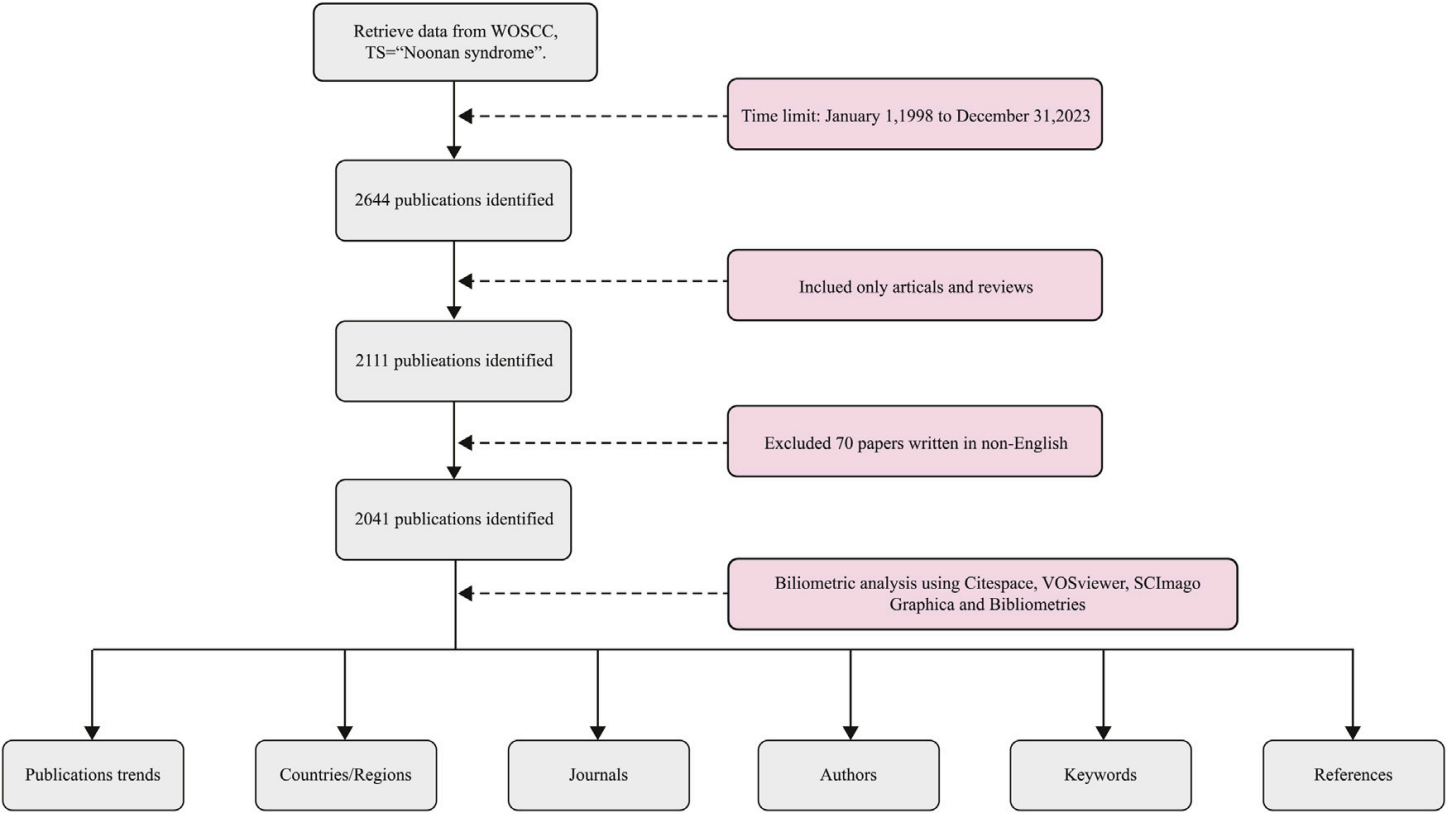
Flowchart of literature search and data analysis.

Web of Science Core Collection (WOSCC) is an essential database for accessing global academic information, which includes international academic journals with high authority and influence, ensures the reliability of the quality of the included literature, and is recognized as the most appropriate database for literature measurement (Jia et al., 2024). Among them, Science Citation Index-Expanded (SCI-E), as a sub-database of WOSCC, includes more than 9,500 high-quality journals in 178 disciplines, including clinical medicine (Tang et al., 2024). To prevent statistical bias due to database update, we completed the literature search and data download from WOSCC (SCI-E) on 27 July 2024, with the following search requirements: (1) search subject: TS=(“Noonan syndrome”); (2) The article types were limited to “artical” and “review”; (3) The screening language was “English”; (4) The time range was limited to 1998-01-01 to 2023-12-31. The detailed search flowchart is shown in Figure 1. Two authors (CUI and LUO) independently collected and compared all the data, with an agreement rate of 0.96. In the end, a total of 2,041 articles that met the requirements were collected for inclusion in this study.

### 2.2 Data analysis and visualization

We used Citespace (6.3.R1) (Chen, 2006) and VOSviewer (1.6.19) (Kirby, 2023) to analyze the co-occurrence, co-citation, and burst detection for institutions, authors, keywords, and references, respectively, to obtain evaluative data such as the number of publications, centrality, co-citation frequency, etc. We plotted the main results in order to show the information on NS publications. The settings of Citespace usage are as follows: The period is 1998–2023, the time node is set to 1 year, the node type is selected as Country, Keyword, etc., the node strength defaults to Cosine, the threshold is selected as TOP50, and the network cropping ribbon is selected as pathfinder algorithm for the mapping analysis. When using VOSviewer to analyze, select “data type from WOS,” set the counting method to Full counting, add a word list to clean the data, and then set the minimum frequency of occurrence of the words to form a graph that meets the requirements finally. Scimago Graphica (1.0.34) (Hassan-Montero et al., 2022) and BibliometrixR (Kratz et al., 2005) have potent data visualization capabilities and were used in this study to demonstrate the development of inter-country collaboration and journals in the field of NS. Excel software was also utilized in the study to tabulate various data and present the primary data in a table in the text.

## 3 Results

### 3.1 Trends in the number of publications

**FIGURE 2:**
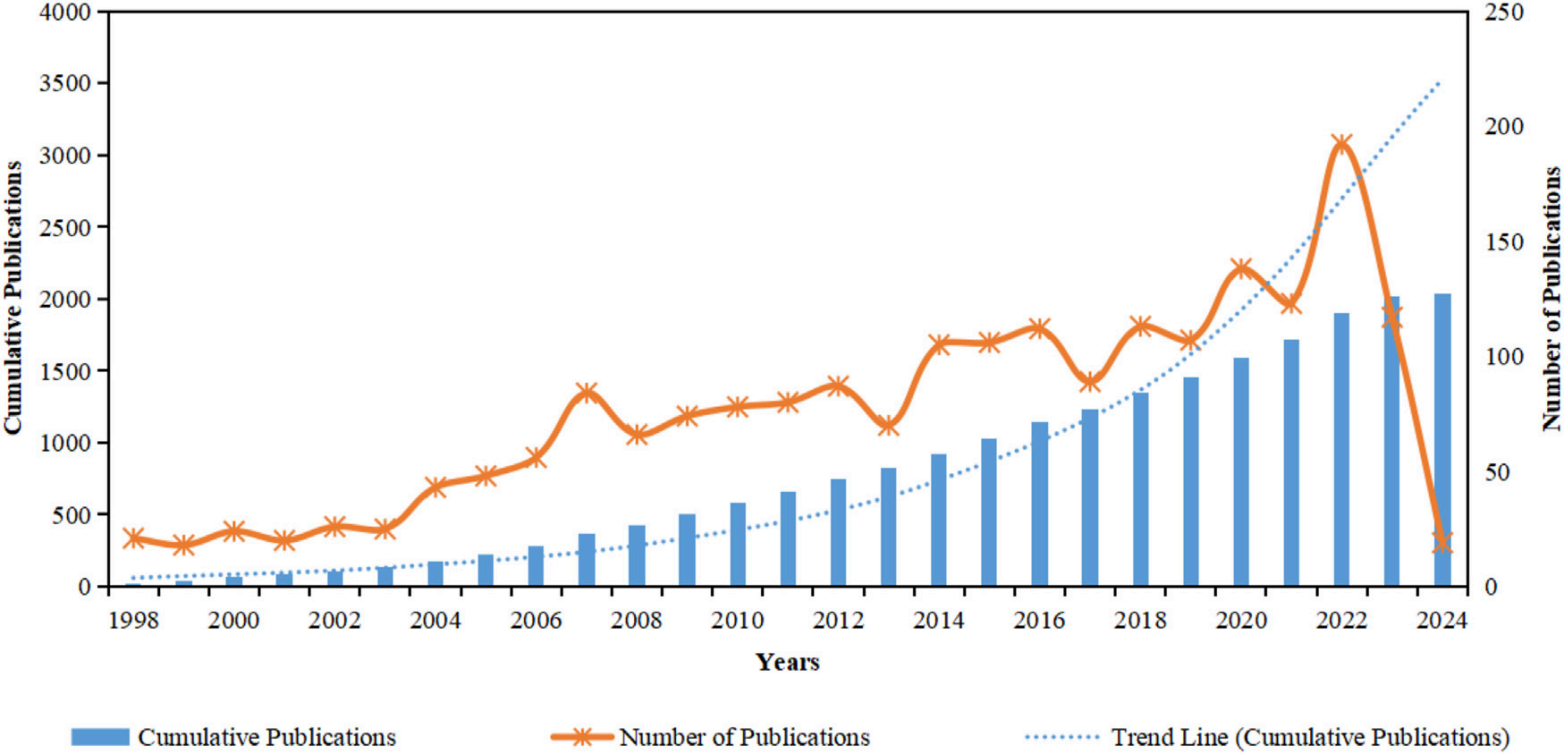
Statistics on issuance of publications.

After several preliminary search tests, we found that taking 1998 as the starting year of the search not only could collect a sufficient number of publications but also could accurately reflect the recent overview of NS research, making the study comprehensive and not losing precision. In the past 26 years, a total of 2041 NS-related publications have been published in various high-quality journals, and the cumulative number of publications is represented by the blue bars in Figure 2, with a clear upward trend line of growth and the total number of publications has a great potential in the future. The annual number of publications corresponds to the orange markers. We have divided them into three stages of development according to the different growth trends: (1) The “research start-up period” appeared from 1998 to 2007, and the number of publications gradually increased from the lowest value, and a certain amount of research results have been accumulated. (2) The “stable development period” was characterized by moderate growth between 2008 and 2016, which increased the attention and awareness of NS. (3) The “fluctuating upward period” includes the period from 2017 to 2023, during which three peaks in the number of articles were seen and a historical breakthrough was achieved. The year 2022, in the third phase, realized the highest value of historical publications (n = 192), which is 10.6 times higher than the lowest value in 1999 (n = 18) in the first phase. Overall, NS has been fruitful and continues to increase, and the growth rate is not expected to slow down in 2024.

### 3.2 Analysis of countries/regions and institutions

**FIGURE 3:**
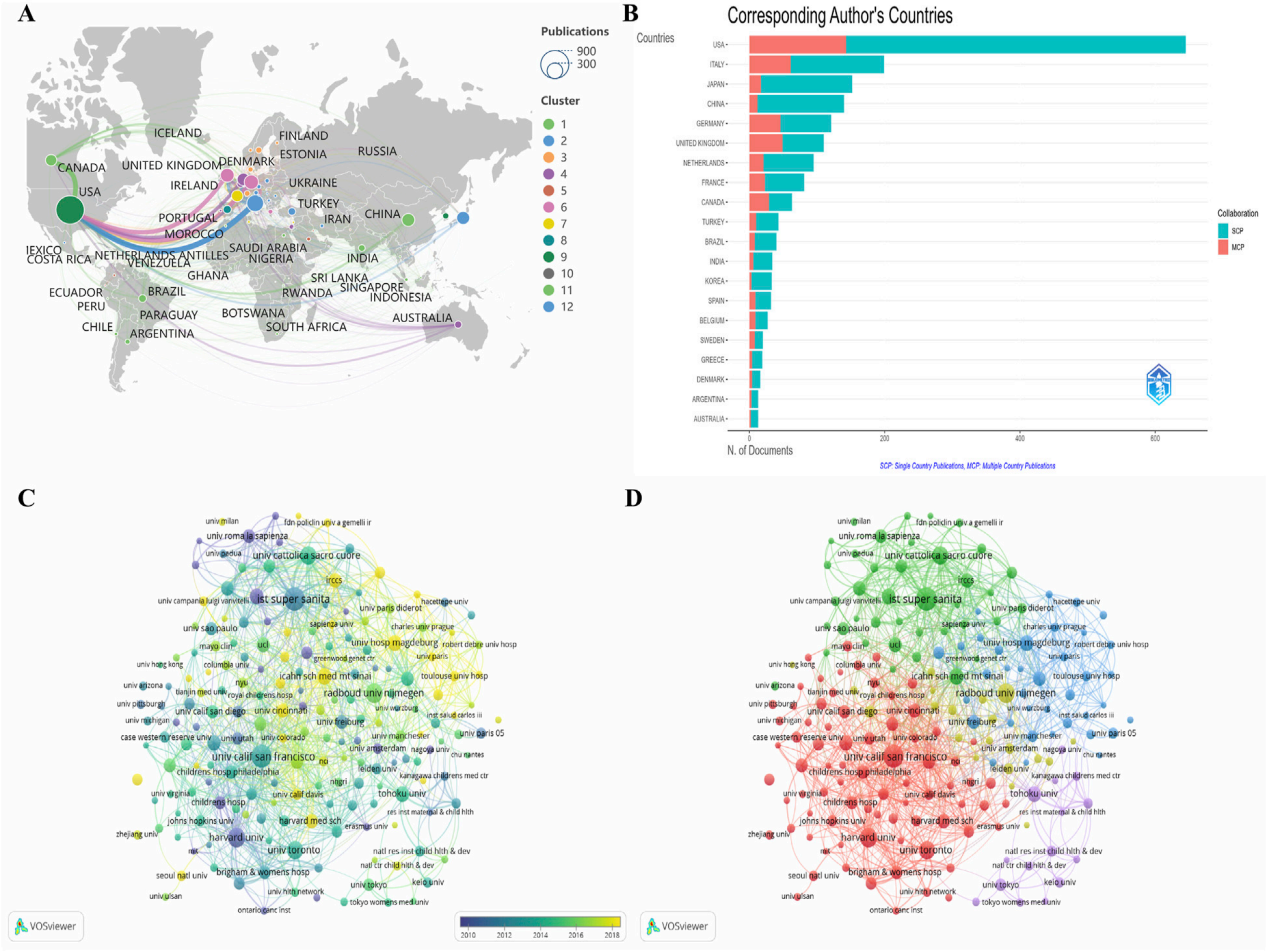
Visualization map of NS publication countries/regions and institutions. **(A)** Global bibliometric mapping. **(B)** Map of domestic and foreign collaborations. **(C)** Time-ordered visualization of institutions. **(D)** Institutional collaboration network mapping.

A total of 61 countries/regions and 2,173 organizations participated in NS research. Table 1 lists the ten most published countries, which produced 80.8% of the research output, with United States, ITALY, and JAPAN topping the list with 645, 199, and 152 articles, respectively. The United States had the highest number of total citations, and CANADA had the highest number of citations per publication. The global distribution of publications (Figure 3A) shows the collaboration between the significant publishing countries/regions at a macro level, with the United States collaborating most closely with CANADA and the European countries.MCP stands for articles collaborated by multiple countries, while SCP stands for single-country articles. Figure 3B shows that CANADA and UNITED KINGDOM have the highest percentage of MCPs, followed by GERMANY and ITALY. The top ten institutions in terms of the number of publications are shown in Table 2, with United States and ITALY having the most significant percentage of institutions. Istituto Superiore di Sanità (n = 79) leads in terms of the number of publications and total citations (n = 9,184), and also in terms of the number of publications is the University of California, San Francisco (n = 73) and Radboud University (n = 54). Figure 3C is chronologically sorted with a gradual change in color, where yellow nodes can be considered as emerging major research institutions. The collaborative network in Figure 3D consists of five clusters, each with a central node located in the top ten in terms of the number of publications, which are firmly connected, and the institutions with the highest TLS are Istituto Superiore di Sanità and Università Cattolica del Sacro Cuore.

**TABLE 1:** The 10 countries/regions with the most publications in the field of NS.

Rank	Country/Region	Publications	MCP	Citations	Citation per publication
1	United States	645 (31.60%)	143 (22.2%)	36316	56.3
2	Italy	199 (9.75%)	61 (30.7%)	7,091	35.6
3	Japan	152 (7.45%)	17 (11.2%)	4,805	31.6
4	China	140 (6.86%)	12 (8.6%)	2096	15
5	Germany	121 (5.93%)	46 (38.0%)	6,133	50.7
6	United Kingdom	110 (5.39%)	49 (44.5%)	3,813	34.7
7	Netherlands	95 (4.65%)	21 (22.1%)	2,871	30.2
8	France	81 (3.97%)	23 (28.4%)	2,380	29.4
9	Canada	63 (3.09%)	29 (46.0%)	3,812	60.5
10	Turkey	43 (2.11%)	10 (23.3%)	404	9.4

**TABLE 2:** The 10 institutions with the most publications in the field of NS.

Rank	Institution	Country	Publications	Citations	TLS
1	Istituto Superiore di Sanità	Italy	79	9,184	441
2	University of California, San Francisco	United States	73	8,284	287
3	Radboud University	Netherlands	54	1,506	166
4	Harvard University	United States	54	7,993	236
5	University of Toronto	Canada	49	3,190	138
6	Università Cattolica del Sacro Cuore	Italy	48	4,596	339
7	Tohoku University	Japan	36	2,492	199
8	University Hospital Magdeburg	Germany	36	1,365	265
9	Harvard Medical School	United States	30	793	120
10	Scientific Institute for Research, Hospitalization and Healthcare (IRCCS)	Italy	29	989	186

### 3.3 Analysis of authors and co-cited authors

**FIGURE 4:**
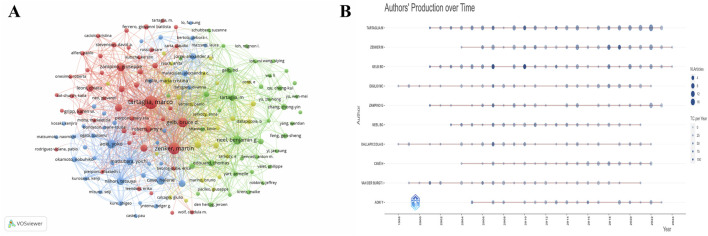
Author’s analysis. **(A)** Visualization of author collaborations. **(B)** Graph of temporal trends in posts by lead authors.

A total of 11,147 authors have been counted to have signed publications in the field of NS during the 26 years Table 3 shows the information of the top 10 productive authors, with TARTAGLIA M contributing 118 publications and 5,147 citations, both in the first place. ZENKER M (n = 82) and GELB BD (n = 76) are in second and third place in the ranking of the number of publications. The “Fractionalized Frequency” is used to specify the authors’ contribution to each article, and the “Local Impact by H index” reflects the productivity and research impact of scholars. The three authors mentioned above, whose contribution rate is more than 10 and whose impact indexes are among the top, are considered to be essential drivers of the forward development of the NS field. Figure 4A shows the visualization of authors and co-cited authors using VOSviewer software; different colors represent different author clusters or teams, the node size is proportional to the number of papers, and the thickness of the lines reflects the strength of the cooperative relationship between authors. It can be seen that there are four main author clusters in the field of NS, among which the red cluster with TARTAGLIA M as the center node, the blue cluster with AOKI Y as the center node, and the green cluster with NEEL BG as the center node constitute an essential support for NS research. Figure 4B chronologically sorts the number of publications and citations by year for the authors listed in Table 1, with DIGILIO MC and DALLAPICCOLA B having the most extended research spans and ZENKER M’s research published in 2019 being the most influential in recent years.

**TABLE 3:** Top 10 most productive authors and co-cited authors.

Rank	Author	Publications	Fractionalized frequency	Local impact	Citation
1	Tartaglia M	118	11.06	45	5,147
2	Zenker M	82	10.71	36	2,334
3	Gelb BD	76	10.55	43	4,805
4	Digilio MC	49	5.44	31	1841
5	Zampino G	46	3.20	25	2,533
6	Neel BG	43	5.58	32	2,129
7	Dallapiccola B	41	3.62	31	2,122
8	Cavé H	40	3.02	22	810
9	Van Der Burgt I	40	6.35	25	2,576
10	Aoki Y	38	4.60	17	1,291

### 3.4 Analysis of core journals

**FIGURE 5:**
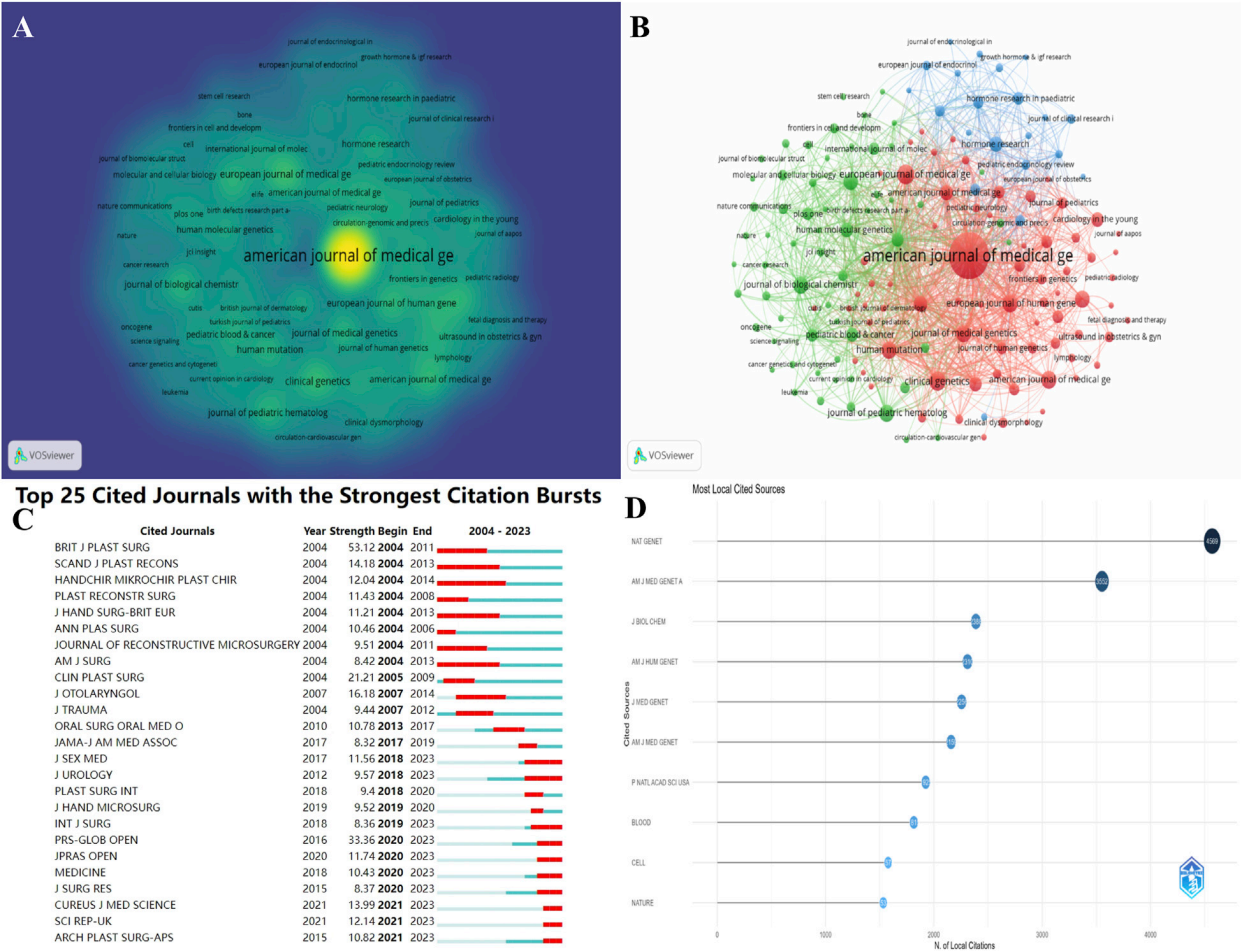
Journal analysis of published NS-related studies. **(A)** Density view of journals. **(B)** Collaborative clustering of journals. **(C)** Top 25 journals with the highest intensity of outbreaks. **(D)** Top 10 most cited journals in NS.

Since 1998, 723 journals have published research results related to NS, and they have made outstanding contributions to the dissemination of knowledge and the advancement of the discipline. However, there is variability in the influence and contribution of each journal in the field. We show the distribution of the number of publications through the journal density plot (Figure 5A), where the brighter color indicates the higher number of publications, among which AMERICAN JOURNAL OF MEDICAL GENETICS is the most prolific journal in the field of NS, with a very significant advantage of publications. However, we learned that it divided its journals into three directions to run them independently after 2003 in order to accurately target journals that have made more contributions to the NS field, and we counted them separately in our statistics. Table 4 organizes the details of the highly productive journals; AMERICAN JOURNAL OF MEDICAL GENETICS PART A, with a total of 178 articles, is the most contributing journal, followed by the EUROPEAN JOURNAL OF MEDICAL GENETICS (n = 32) and CLINICAL GENETICS (n = 30). PROCEEDINGS OF THE NATIONAL ACADEMY OF SCIENCES OF THE United States is the most productive journal with the highest impact factor and is located in the JCR Q1 region. Figure 5B visualizes the collaborations between journals and forms three main collaboration clusters. The results of the outbreak detection analysis of journals are shown in Figure 5C; the journal with the highest intensity is the British Journal of Plastic Surgery (53.12), and Plastic and Reconstructive Surgery - Global Open has an outbreak intensity of 33.36, which is the highest in the last 3 years. Figure 5D ranks the most cited journals, with Nature Genetics (n = 4,569), AMERICAN JOURNAL OF MEDICAL GENETICS PART A (n = 3,552), and Journal of Biological Chemistry (2,388) topping the list with a high impact.

**TABLE 4:** Information on the top 10 journals in terms of publications.

Rank	Journal	Publication	IF (2023)	JCR	Citations
1	American Journal of Medical Genetics Part A	178	1.7	Q3	3,552
2	European Journal of Medical Genetics	32	1.6	Q3	374
3	Clinical Genetics	30	2.9	Q2	797
4	European Journal of Human Genetics	27	3.7	Q2	856
5	American Journal of Medical Genetics	26	3.659 (2004)	Q2	505
6	Journal of Biological Chemistry	25	4	Q2	2,388
7	Journal of Medical Genetics	25	3.5	Q2	2,256
8	Proceedings of The National Academy of Sciences of the United States	25	9.4	Q1	1925
9	Human Mutation	24	3.3	Q2	1,194
10	Journal of Pediatric Hematology Oncology	24	0.9	Q4	344

### 3.5 Analysis of keywords and research areas

**FIGURE 6:**
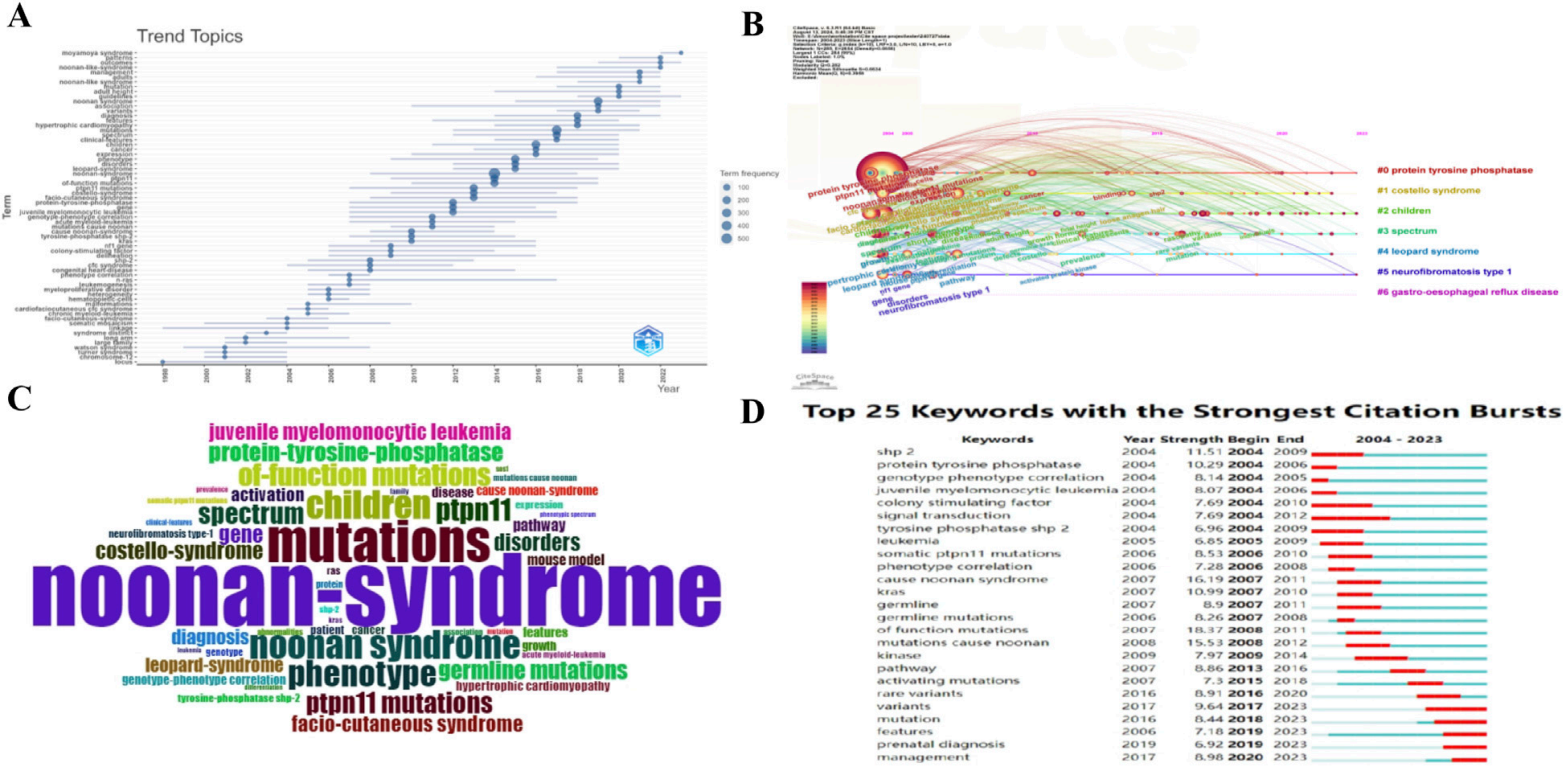
Visualization of NS keywords. **(A)** Keyword-based trends in research topics. **(B)** Timeline of keyword clustering **(C)** Word cloud map of keywords. **(D)** Top 25 keywords with the highest burst intensity.

A total of 3,808 different keywords were collected in this study. We counted the frequency of occurrence of each keyword, and the 20 keywords with the highest number of occurrences were made into Table 5, and they were visualized in Figure 6C noonan-syndrome (n = 594), mutations (n = 344), noonan syndrome (n = 236), children (n = 231), and phenotype (n = 218) are the most popular terms in NS research, illustrating that the child population is the main target of research and that gene mutations and disease phenotypes are widely emphasized in the field of NS. By refining and categorizing the frequency of keywords, we can grasp the research themes in the field, which is an important basis for exploring the research hotspots in the field. Figure 6A was sorted and organized according to the frequency of words appearing in different periods to form a thematic trend map. LOCUS and MOYAMOYA SYNDROME were the earliest and the latest words appearing, respectively. In contrast, the others were mainly based on the genes and various types of related syndromes. By cluster analysis of the keywords, we obtained seven clusters, which are #0protein tyrosine phosphatase, #1costello syndrome, #2children, #3spectrum, #4leopard syndrome, #5neurofibromatosis, and #5neurofibromatosis. 5neurofibromatosis type 1, and #6gastro-oesophageal reflux disease.In the keyword outbreak detection results (Figure 6D), the top three outbreak intensities were of function mutations (18.37), cause noonan syndrome (16.19), and mutations cause noonan (15.53). Although research hotspots can be clarified through these keywords, their outbreaks have ended from 2011 to 2012, indicating that these hotspots have been focused on in the past. We found that five keywords exploded in the last few years and have continued until now: variants, mutations, features, prenatal diagnosis, and management, from which we are expected to find the research frontiers in the field of NS.

**TABLE 5:** Top 20 keywords related to NS.

Rank	Keyword	Counts	Rank	Keyword	Counts
1	noonan-syndrome	594	11	germline mutations	150
2	mutations	344	12	costello-syndrome	139
3	noonan syndrome	236	13	disorders	139
4	children	231	14	gene	139
5	phenotype	218	15	facio-cutaneous syndrome	135
6	ptpn11	185	16	juvenile myelomonocytic leukemia	135
7	of-function mutations	182	17	diagnosis	123
8	spectrum	173	18	leopard-syndrome	117
9	ptpn11 mutations	172	19	activation	114
10	protein-tyrosine-phosphatase	153	20	pathway	97

### 3.6 Analysis of references

**FIGURE 7:**
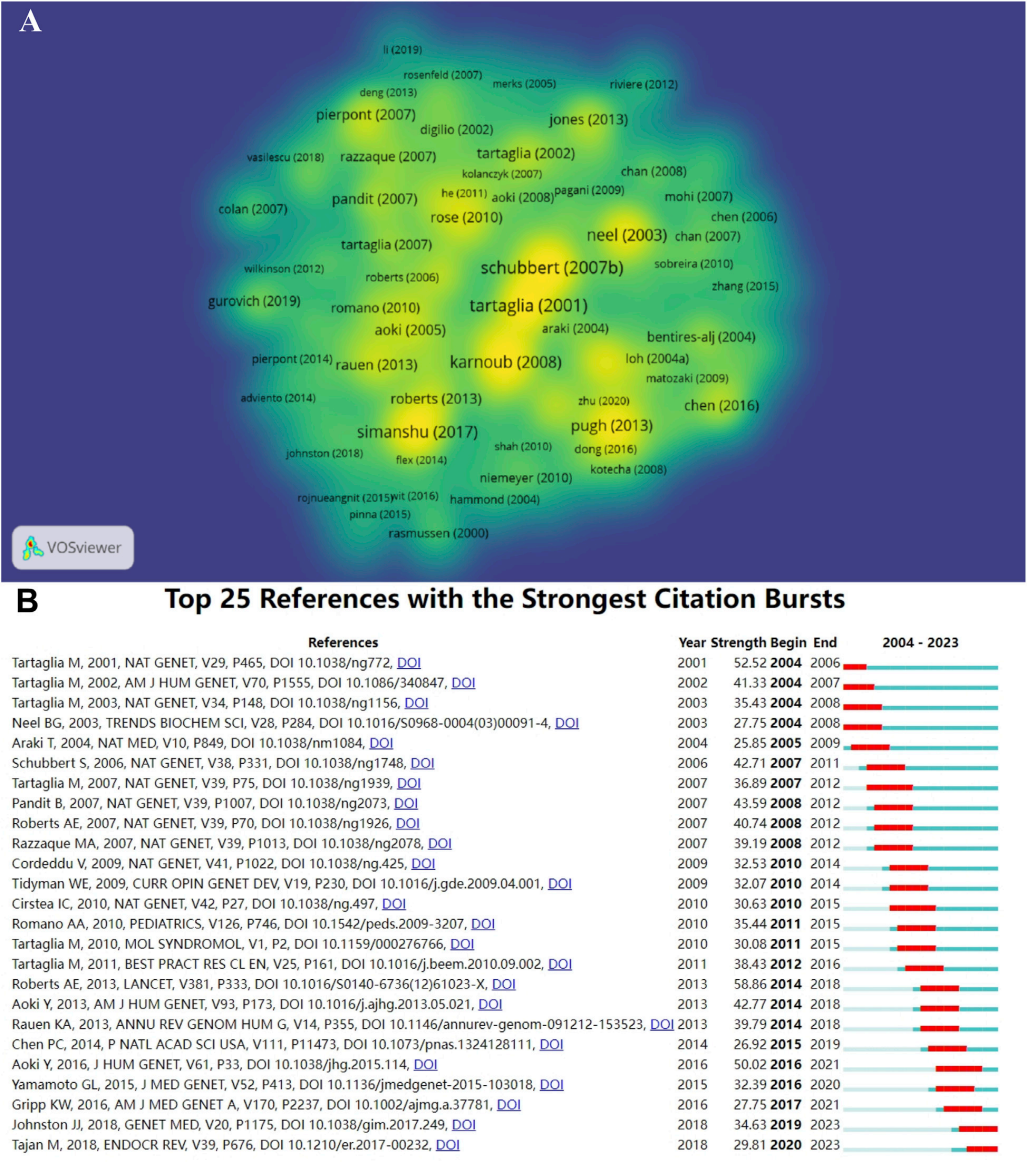
Visualization of references. **(A)** Density plot of the number of citations to the literature **(B)** The 25 citations with the highest intensity of outbreaks.

We plotted the density of citation counts of references (Figure 7A), and the denser nodes in the plot showed a multipolar distribution, suggesting that they collectively guide and lead the relevant research in NS. The information of the 10 most cited papers is tallied in Supplementary Table S1 with the title “Mutations in PTPN11, encoding the protein tyrosine phosphatase SHP-2, cause Noonan syndrome ” (n = 1,244) ranked first in the number of citations and was the most influential article. The mean impact factor of the journals that published these ten papers was 43.75, and the high-quality citations provided a theoretical foundation for NS research. In Figure 7A, (Tartaglia et al., 2001), high explosive citations are integrated, with the intensity concentrated between 26.92 and 58.86, among which “Roberts AE, 2013, LANCET, V381, P333, DOI 10.1016/S0140-6736 (12)61023-X” has the most explosive and is the hot literature that is co-opted. Each citation’s outburst lasted three to 5 years, reflecting the progress of research and updating of results. There were two citations in an outburst state in 2023, which reflects current research priorities.

## 4 Discussion

NS was first discovered and reported by Jacqueline Noonan in 1968. After more than 50 years of intensive research, it is now generally accepted that the typical features of NS can be summarized as unique facial features, congenital heart disease, short stature, and developmental delay, but clinically, NS is heterogeneous, with a wide variation in the severity of symptoms (Miller, 2019). It has been reported that 50%–80% of patients with NS are associated with congenital heart disease, which has become the second leading cause of the disease after trisomy 21, posing a significant threat to human life and health (Busley et al., 2024). In addition, it also widely affects the endocrine system (De Schepper et al., 2024), reproductive system (Orsolini et al., 2024), nervous system (Samuels and Northrup, 2024), blood system (Barg et al., 2024), skeletal system (Miura et al., 2001), etc., causing irreversible harm to children’s growth and development and aggravating the economic and psychological burdens of patients’ families. With the advent of molecular diagnostic techniques, NS patients can be diagnosed at an early stage. They can be used to exclude other similar diseases, which has dramatically improved the diagnosis rate of NS. However, the treatment of NS is still a significant challenge for the medical community. Therefore, it is crucial for human health that the field of NS be fully recognized and continuously explored.

NS-related research has achieved fruitful results in the past 26 years. Although the annual publication volume has fluctuated up and down, the overall growth trend is evident, suggesting that the field of NS has an excellent prospect for sustained research in the future. Especially in the third phase, the number of annual publications has a unique pattern to follow, showing apparent fluctuations and upward momentum, which we analyze may be the result of the combined influence of three factors: the lag in the publication of papers, the synchronization of the implementation of significant topics, and the iterative nature of the research progress of the researchers. This phenomenon is common to every field in the early stage of booming development, and NS-related research is expected to exceed 200 papers for the first time in 2024. With the development and popularization of genetic testing technology, more and more scientists are focusing on the field of NS. The number of literature will surge in the next stage, but this does not mean that all regions of the world have joined the research team of NS, so we conducted an in-depth analysis of the article’s country/region, institution, and authors in an attempt to find the differences and promote the joint research in the field of NS.

The countries/regions that have made outstanding contributions to NS research are mainly concentrated in North America, Europe, and East Asia, with close to one-third of the results coming from the United States, which has the closest cooperation with European countries, strongly contributing to the high-quality development of NS research. These high-yielding countries mainly belong to developed countries, and we believe that the main reason is that the medical technology in these countries/regions is at an advanced level, which helps the accurate diagnosis and intervention of NS, and in addition, the affluent material conditions make people pay more attention to NS. Although China is still a developing country, its GDP is the second largest in the world, and its medical treatment and living standards have been greatly improved.MCP reflects the ability of academic exchanges between countries, and CANADA and UNITED KINGDOM are the models of global cooperation in the field of NS. Although JAPAN and CHINA have outstanding publication volumes, their MCP is low, and they need to continuously develop an international perspective and promote the formation of East Asian regional cooperation. The geographical distribution of the top ten institutions in terms of the number of publications is consistent with the pattern followed by the countries, with the most significant proportion of institutions from the United States and Italy, among which the Istituto Superiore di Sanità has not only the highest number of citations but also the highest total connectivity intensity, making it the most influential institution in the field of NS. Secondly, Università Cattolica del Sacro Cuore collaborates more frequently with other institutions around the world. The University of California publishes research results that lead in both quality and quantity, and they are both leading institutions in NS research. The emerging research institutions represented by the yellow nodes in Figure 3C should take the cluster as a carrier to broaden the channels of cooperation continuously, connect with institutions with strong influence through project cooperation and personnel exchange, and develop into a larger node to drive and promote more new progress in the field of NS.

The NS field formed a sizeable cluster of author collaborations, but the development was uneven among the different clusters. TARTAGLIA M, ZENKER M, and GELB BD are considered to be the most authoritative NS scientists, and they are all in the same cluster, which makes this cluster significantly more potent as a whole.2023 Together, they have utilized three-dimensional cardio bodies and bio-artificial cardiac tissues to reveal the mechanistic link between NS-associated RAF1 (S257L) and severe hypertrophic cardiomyopathy (Nakhaei-Rad et al., 2023). In contrast, there are clusters that authors do not lead with larger nodes, producing polarization between clusters. We believe that critical researchers in the field of NS should not only maintain close collaborative relationships within the clusters but also take the initiative to strengthen communication with authors outside the clusters to expand the influence of the clusters and, at the same time, drive the overall development of global NS research. Based on the authors’ yearly publication clues obtained in the results section, we targeted a study by ZENKER M. It is the latest highly cited paper, which proposes a facial image analysis framework, “DeepGestalt,” which quantifies the similarity of hundreds of syndromes and genetic disorders using computer vision and deep learning algorithms, and not only can it quantify the similarity between the syndromes and the genetic disorders, but also can help the researchers to improve the quality of their work (Gurovich et al., 2019).

The significant journals publishing NS results are concentrated in the field of genetic research, which both highlight the attribute that NS belongs to the genetic class of diseases and reflect the disciplinary inclination of the journals issuing articles. Although AMERICAN JOURNAL OF MEDICAL GENETICS PART A occupies an obvious advantage in the number of articles published, the impact factor is relatively low, which may be attributed to the short time of the journal’s founding and the rapid growth of the number of articles published. In the future development of the journal, it should strengthen the construction of academic quality and enhance the academic impact of NS-related research. PROCEEDINGS OF THE NATIONAL ACADEMY OF SCIENCES OF THE United States, as a high-quality journal that publishes a large number of NS papers, should select and disseminate more high-level research results and promote the discipline to realize leapfrog progress. The high-yield journals, high-outbreak journals, and highly cited journals identified in this study are the mainstay of the expanding field of NS, and they have to play a leading role in the cluster to achieve simultaneous improvement in quantity and quality. The latest outbreak journals, on the other hand, are the reserve force, which may be insignificant in terms of impact but can attract and receive more meaningful results by adopting innovative publication modes.

Digging deeper into the research content and direction of the references helps to gain a comprehensive understanding of the hot topics of NS. In this study, we identified the most influential references based on citation frequency, which have played an essential role in laying the foundation for scholarship in the field Tartaglia et al. (2001) first found that missense mutations in PTPN11 can cause NS and pointed out that the pathogenesis of this disease is caused by excessive non-receptor-type protein tyrosine phosphatase activity, which has pioneered the study of the gene of NS and has become an indispensable citation in subsequent NS research. An indispensable citation in subsequent NS research. Since the pathogenic genes associated with NS encode essential proteins in the RAS-MAPK signaling pathway, most of the subsequent highly cited references are in-depth studies or retrospective analyses of this pathway (Schubbert et al., 2007; Karnoub and Weinberg, 2008; Simanshu et al., 2017). The comprehensive understanding of NS published in the journal LANCET has received extensive and sustained scholarly attention (Roberts et al., 2013). The clinical and genetic information on the autosomal recessive genes and RASopathies of NS has become a hot topic in the recent past (Johnston et al., 2018; Tajan et al., 2018). These important references are not up-to-date in terms of time of publication, but they reflect emerging themes in the phase of NS research.

Through the comprehensive summarization of NS research’s keyword frequency, clustering, outbreak intensity, and topic timeline trend, we summarized that we got the hot topics during the past 26 years, focusing on the correspondence between NS genotypes and phenotypes and the precise diagnosis of NS. The future frontier direction is the long-term intervention strategy of NS. At present, scientists have found that mutations in more than 20 genes are associated with the pathogenesis of NS, specifically including PTPN11, SOS1, SOS2, KRAS, NRAS, RIT1, RRAS, RASA1, RASA2, MRAS, RAF1, BRAF, MAP2K1, MAP3K8, SHOC2, PPP1CB, SPRY1, LZTR1, MYST4, A2ML1, SPRED2, and CBL, etc., of which LZTR1 and SPRED2 are inherited in autosomal recessive inheritance (Chen et al., 2024). PTPN11 is localized in the region of chromosome 12q24.1, and it mainly affects growth factors and cytokines through the regulation of the RAS-MAPK signaling pathway by SHP-2 proteins, which is mainly manifested as short stature and skeletal malformations (Jorge et al., 2022). It also has a high incidence of pulmonary vein stenosis, atrial septal defect, and cryptorchidism (Pannone et al., 2017). SOS1 gene is located in chromosome 2p22-p21 region, which encodes for the production of RAS-specific guanine nucleotide exchange factor and induces active RAS-GTP to act on the RAS-MAPK signaling pathway, which affects ectoderm mainly in NS. However, mental retardation is less frequent than PTPN11 (Sigamani et al., 2021). The RAF1 gene is located in the chromosome 3p25 region, and three species of RAF can activate the MEK-ERK cascade to produce RAS effects, which are manifested as prominent hypertrophic cardiomyopathy (Zheng et al., 2024). Mutations in the KRAS gene in the 12p12.1 region of chromosome 12p12.1 showed significant abnormalities in cognitive function (Razzaque et al., 2012). SHOC2 gene in chromosome 10q25 region upregulates the RAS-MAPK pathway and is characterized by alopecia, hyperactivity, mitral valve dysplasia, and atrial septal defect in NS (Wilson et al., 2021). CBL, RIT1, and BRAF are located in chromosome 11q23.3, 1q22, and 7q34 regions. Individuals with CBL mutations have specific manifestations of left atrial enlargement, mitral valve, aortic valve stenosis, mitral valve closure insufficiency, and susceptibility to juvenile granulomonocytic leukemia (Niemeyer et al., 2010; Pérez et al., 2010). RIT1 mutations are associated with a higher incidence of hypertrophic cardiomyopathy (Leegaard et al., 2022). BRAF mutations, although closely associated with cardio-facial-cutaneous syndrome, are characterized by the typical features of NS without the manifestation of cardio-facial-cutaneous syndrome in the carriers (Sarkozy et al., 2009). The above are the common NS genotypes. These are the results of common NS genotype-phenotype relationships, which need to be explored more comprehensively by scientists in order to improve the accuracy of clinical diagnosis and the prospective of intervention. Currently, the diagnosis of NS is categorized into phenotypic diagnosis, molecular diagnosis, and prenatal diagnosis. The most commonly used diagnostic criteria for clinical diagnosis (Vanderburgt et al., 1994) are those proposed by Dutch scholar van der Burgt in 1994, which categorize facial features (typical peculiar features/specific features), heart ([PVS, HCM, typical ECG changes]/other heart defects), height (smaller than the 3rd percentile of the same age and sex/10th percentile), thoracic profile (chicken chest or funnel chest/wide thoracic profile), family history (first-degree relative diagnosed/proposed NS) and other (concurrent/non-concurrent symptoms: IQ backwardness, cryptorchidism, and lymphatic dysplasia) were categorized into major and minor criteria, with only one of the other major or two of the other minor criteria being fulfilled if the patient had typical facial features, and two of the other major or three of the other minor criteria being fulfilled if the patient had atypical features to make the diagnosis of NS. In female patients with NS phenotypes, Turner syndrome cannot be excluded by clinical diagnosis alone, and karyotyping is an option (Siqueiros-Sanchez et al., 2024). Genetic testing is currently the most popular technique, and it is generally accepted that PTPN11 is preferred for genetic screening because variants in this gene explain the greatest number of cases. Then, the corresponding genotype is selected as the next screening target based on the patient’s phenotypic characteristics. Of course, a negative result obtained by genetic testing cannot completely exclude the diagnosis of NS, as there is still a longer way to go before all genotypes of NS are found. Prenatal diagnosis is necessary if the patient carries a clear pathogenic variant. Chorionic villus aspiration and amniocentesis are chosen to obtain fetal samples and analyze fetal DNA for variants in the early and middle trimesters of pregnancy, respectively (Margiotti et al., 2024). After a definitive diagnosis of NS, a thorough evaluation of all systems is essential, and although there is no specific treatment for NS, scientists are constantly striving to improve the quality of life of patients. In patients with NS, the progression of primary cardiomyopathy with autosomal dominant inheritance is more unknown, which can be self-resolving in mild cases or life-threatening in severe cases and should be followed up promptly and given pharmacologic interventions such as β-blockers (Yi et al., 2022). The U.S. Food and Drug Administration (Grimberg and Hawkes, 2024) recommends recombinant human growth hormone (rhGH) for the treatment of NS-induced short stature. However, it is controversial because of the risk of HCM and tumors that exist in some NS genotypes after treatment. More in-depth studies on the association between rhGH therapeutic dose, genotype, insulin-like growth factor 1 levels, and rhGH treatment effects are also needed in the future (Wu et al., 2023). Timely treatment with hearing aids or cochlear implants for those with hearing loss can significantly improve the quality of life for patients (Gao et al., 2021). We believe that genetic counseling is superior to all treatments and can promote eugenics, especially in identifying and guiding parents with gonadal chimerism. Researchers have found new clues to treating NS in their ongoing attempts and explorations, a discovery that will bring light to patients with NS. RASopathies are collectively referred to as a series of genetic syndromes caused by mutations in the genes involved in the RAS/mitogen-activated protein kinase pathway (Rauen et al., 2013). NS is recognized as one of the disorders. However, the drugs developed and approved based on specific components of this pathway are mainly applied in cancer treatment and less in other diseases. In recent years, a growing body of research has suggested that targeting MEK inhibitors (MEKis), initially developed for cancer therapy, to intervene in mutational endings occurring in RASopathies is a promising therapeutic approach (Gazzin et al., 2024; Saint-Laurent et al., 2024). It has been shown that exposure of developing embryos to an appropriately tolerated dose of MEKis (PD0325901) reduces developmental defects typically seen in K-RasV14I mice (Hernández-Porras et al., 2014). One study (Wu et al., 2011) of MEK inhibition in postnatal L613V/+ mice found normalization of developmental, facial, and cardiac defects, suggesting that MEKis could be a potential therapeutic agent for the treatment of NS. In a case report (Leegaard et al., 2022), children with NS with the RIT1 variant developed premature HCM and right ventricular outflow tract (obstruction), leading to a poor prognosis, and the physician intervened with MEKis trametinib over-the-counter with a more satisfactory outcome. However, due to the low prevalence of NS and the fact that this treatment option needs to be formally approved, there is a need for large-sample, multicenter clinical trials to validate its efficacy. We believe that future interventions in NS using new drugs such as MEKis will be the focus of researchers’ attention, including their optimal therapeutic window, optimal dosage, long-term adverse effects, and impact on other comorbidities in NS, which will need to be shared and promoted by scholars worldwide.

## 5 Advantages and limitations

This is the world’s first bibliometric analysis of high-quality results in the field of NS. This study comprehensively analyzes the information of countries, institutions, authors, and journals included in the papers. It identifies the hot topics and cutting-edge directions in the field of NS, which lays the foundation for the future development of the discipline. Admittedly, there are some limitations in this study: we only included papers from the WOSCC database and only retained the “article” and “review” types of studies in English. However, these limitations emerged under conditions of increased rigor and precision and did not adversely affect the bibliometric findings of NS. Just as we set the final date of the search at 31 December 2023, rather than mid-2024, this ensures that the number of publications between years is comparable and complete, and that precision is not missing in the pursuit of including studies that are comprehensive.

## 6 Conclusion

This study uses bibliometric methods to deeply analyze and visualize NS-related publications in the last 26 years, fully interpreting and visualizing the main contributors to NS research, the current status of global collaborations, and the direction of future trends, which is expected to open up new horizons of high-quality development. Researchers have paid continuous attention to both the correspondence between NS genotypes and phenotypes and precise diagnosis and have achieved fruitful results. Nowadays, the long-term intervention strategy of NS has become a new research goal, and we need to participate together in the future to protect human health with fruitful results.

## Data Availability

The original contributions presented in the study are included in the article/Supplementary Material, further inquiries can be directed to the corresponding author.

## Abbreviations

WOSCC, Web of Science Core Collection; NS, Noonan syndrome; RAS-MAPK, Regulators of the mitogen-activated protein kinase; rhGH, recombinant human growth hormone; MEKis, MEK inhibitors.
